# *In vitro* genotoxicity of nitroimidazoles as a tool in the search of new trypanocidal agents

**DOI:** 10.1590/0074-02760190017

**Published:** 2019-06-27

**Authors:** Ana Claudia Manoel Von Trompowsky, Taline Ramos Conde, Renata Calil Lemos, Bruna Maria CS Quaresma, Marcelly Cristina SR Pitombeira, Alcione Silva de Carvalho, Núbia Boechat, Kelly Salomão, Solange Lisboa de Castro, Helena Pereira da Silva Zamith

**Affiliations:** 1 Fundação Oswaldo Cruz-Fiocruz, Instituto Nacional de Controle de Qualidade em Saúde, Departamento de Farmacologia e Toxicologia, Rio de Janeiro, RJ, Brasil; 2 Fundação Oswaldo Cruz-Fiocruz, Instituto Nacional de Controle de Qualidade em Saúde, Programa de Pós-Graduação em Vigilância Sanitária, Rio de Janeiro, RJ, Brasil; 3 Universidade Federal do Rio de Janeiro, Instituto de Ciências Biomédicas, Programa de Pós-Graduação em Farmacologia e Química Medicinal, Rio de Janeiro, RJ, Brasil; 4 Fundação Oswaldo Cruz-Fiocruz, Instituto de Tecnologia em Fármacos, Departamento de Síntese de Fármacos, Farmanguinhos, Rio de Janeiro, RJ, Brasil; 5 Fundação Oswaldo Cruz-Fiocruz, Instituto Oswaldo Cruz, Laboratório de Biologia Celular, Rio de Janeiro, RJ, Brasil

**Keywords:** genotoxicity, mutagenicity, megazol, nitroimidazoles

## Abstract

**BACKGROUND:**

Only benznidazole (Bnz) (1) and nifurtimox (Nfx) (2) are licensed for the treatment of Chagas disease although their safety and efficacy profile are far from ideal. Farmanguinhos from Fiocruz has developed seven nitroimidazole compounds (4-10) analogs of megazol (3).

**OBJECTIVES:**

To evaluate whether the genotoxic effect of 3 was abolished in the seven nitroimidazoles (4-10) analogs using the *in vitro* alkaline comet assay (CA) and the *in vitro* cytokinesis-block micronucleus assay (CBMN) in whole human blood cells (WHBC) and correlate this effect with their trypanocidal activity using bloodstream trypomastigote forms of *Trypanosoma cruzi*.

**METHODS:**

The toxicity of 3-10 to WHBC in the *in vitro* CA was determined using the fluorescein diacetate/ethidium bromide assay. DNA damage in the *in vitro* CA was evaluated according to tail size in four classes (0-3) and methyl methane-sulfonate (MMS) was used as a positive control. The cytotoxicity of 3-10 to WHBC in the CBMN was measured using the cytokinesis-block proliferation index and the replication index. The number of the micronucleate cells in 2,000 binucleate cells by experimental group was determined. Mitomycin C and N-deacetyl-N-methylcolchicine were used as positive controls.

**FINDINGS:**

Compound 3 showed a significant DNA strand break effect through the *in vitro* CA and highly significant clastogenic and/or aneugenic effect in the CBMN. Compounds 5, 6, 8, 9 and 10 showed negative results in the CBMN and positive results in the *in vitro* CA, while the inverse effect was observed for 4 and 7.

**MAIN CONCLUSIONS:**

Compound 10 was the most promising to proceed with the development as a drug candidate in the treatment of Chagas disease showing absence of chromosomal cytogenetic damage and high activity against *T. cruzi*, about two times higher than 3 and the clinical drug 1.

Chagas disease caused by the protozoan *Trypanosoma cruzi* remains a major social and public health problem in Latin America and is regarded as a neglected tropical disease by World Health Organization (WHO). WHO estimates 5-7 million people are infected with *T. cruzi* worldwide, mainly in Latin America highlighting Argentina, Brazil, Mexico and Bolivia.^1^ In the last two decades, cases have been found in European countries, Japan, Australia and the USA, resulting from the immigration of infected individuals.^1^

Only two drugs, the 2-nitroimidazole benznidazole (Bnz) (1) and the 5-nitrofuran nifurtimox (Nfx) (2) (Fig. 1), are licensed for the treatment of Chagas disease, although their safety and efficacy profile are far from ideal.^2^ Treatment with these drugs is always recommended for all patients in acute phase, in case of accidental contamination with sharp-cutting and contact with mucous membranes, congenital Chagas disease, infected mothers of childbearing age, transfusion-related transmission, reactivated infections in immunosuppressed hosts and chronic disease in children younger than 12 years.^3^ Both drugs have shown successful results and the parasitological cure of treatment with Bnz, occurs in estimated 80% to 100% of patients during the acute phase, but the effectiveness decreases with advancement of the infection; and the data on adults with late chronic infection indicates serological cure only in 5-20% of cases.^4^ The frequency of adverse effects with Nfx is 43.0-97.5% in adults with chronic infection, leading to the discontinuation of the treatment in 14-75% of cases.^5^ Compound Bnz is generally preferred over Nfx because of its better tolerability profile, tissue penetration, and, possibly, efficacy. The most common adverse events associated with Bnz are skin rash (29-50%), digestive intolerance (5-15%), and general symptoms such as anorexia, asthenia, headache, and sleep disturbances (40%) leading to discontinuation of treatment in 9-29% of cases.^6^ The Laboratory of the State of Pernambuco (Lafepe) is the only public laboratory worldwide with authorisation by the Brazilian Health Regulatory Agency (ANVISA) for the production of Bnz since June 2016.^7^

**Fig. 1:**
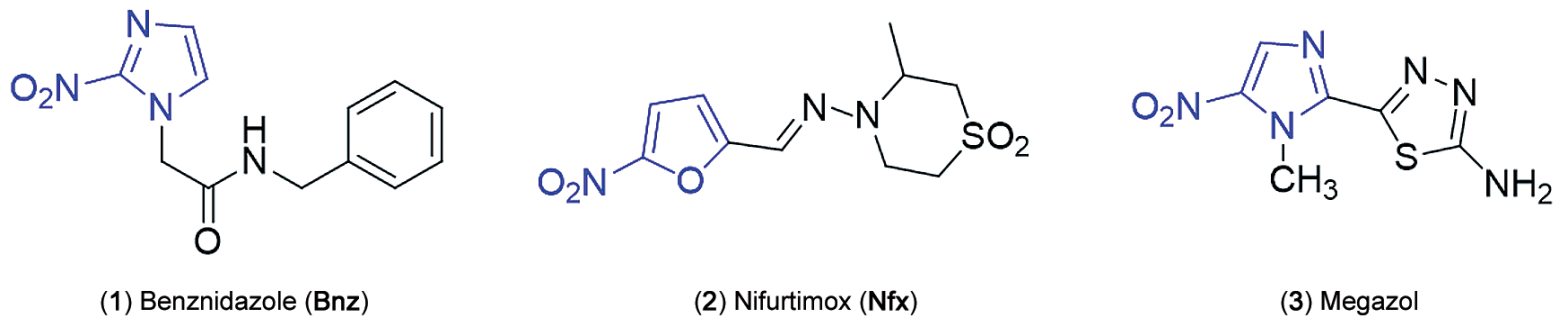
chemical structures of nitroheterocyclic compounds: Benznidazole (Bnz) (1), Nifurtimox (Nfx) (2) and Megazol (3).

Megazol (3) (Fig. 1) is a 5-nitroimidazole with 1,3,4-thiadiazole which exhibits potent *in vitro* and *in vivo* activity against *T. cruzi*, including strains resistant to Bnz, and also *Trypanosoma brucei*, which causes sleeping sickness,^8^ ^,^ ^9^ ^,^ ^10^ ^,^ ^11^ ^,^ ^12^ ^,^ ^13^ which may be due to the triggering of oxidative stress by the compound.^9^ ^,^ ^14^ Compound 3 shows a superior profile of action when compared to Nfx and by the employment of lower doses.^14^ However, it is not used clinically because it has mutagenic activity^11^ ^,^ ^15^ ^,^ ^16^ ^,^ ^17^ associated with the reduction of the nitro group located at position 5 of the nitroimidazole ring.^18^ In view of this, several researchers have been using 3 as a prototype to search for new bioactive substances with high trypanocidal activity^19^ ^,^ ^20^ ^,^ ^21^ ^,^ ^22^ but without the toxic effect.^23^

Nitroimidazoles, as well as nitro compounds in general, have been the subject of discussion about their mutagenicity since their biological activity seems to be related to the damage caused by the bioreduction products of the nitro group to DNA. Previous work of our group showed that the nitro group is not the sole responsible for the genotoxic activity.^24^ The type and position of different substituents bonded to the imidazole ring have a significant influence on the toxicological activity. Continuing our work in the search for bioactive substances and knowing that mutagenicity is an undesirable property in clinically used drugs because raises the question of their potential carcinogenicity, more studies are needed for complete evaluation of nitroimidazole effect on DNA, contributing to the elucidation of mechanisms involved in these processes. A nitroimidazole possessing high trypanocidal activity with none mutagenicity is of great interest not only from a safety point of view, but also provides a basis for further investigations of the mode of action and mechanism of expression of mutagenicity of this class of compounds.^17^

In this article we investigate seven nitroimidazole compounds (**4-10**) analogs to 3^22^ (Fig. 2) using biososterism among rings in order to elucidate the relationship between chemical structure, trypanomicidal and genotoxic activities.^19^ ^,^ ^21^ ^,^ ^22^ In compound 4, the 1,3,4-thiadiazole nucleus presents in 3 was replaced by the 1,2,4-triazole nucleus^22^ (Fig. 2); in 5 in addition to replacing the 1,3,4-thiadiazole nucleus of 3 by the 1,2,4-triazole nucleus with a CF_3_ substituent, the position of this nucleus from the C-2 to the C-5 of the imidazole nucleus was changed with the nitro group at the 4-position, plus NH as a spacer group^19^ (Fig. 2); in 6, the nitro group was transferred from the 5-position of the imidazole ring of 3 to the 4-position of 6 and the 1,3,4-thiadiazole ring of 3 replaced by the pyrazole ring in the α-position with the nitro group.^19^ Ring bioisosterism was also performed to obtain 7, 8, 9 and 10 replacing the pyrazole ring by another azoles with addition of hydrophilic and lipophilic groups respectively, as described in Fig. 2.

**Fig. 2:**
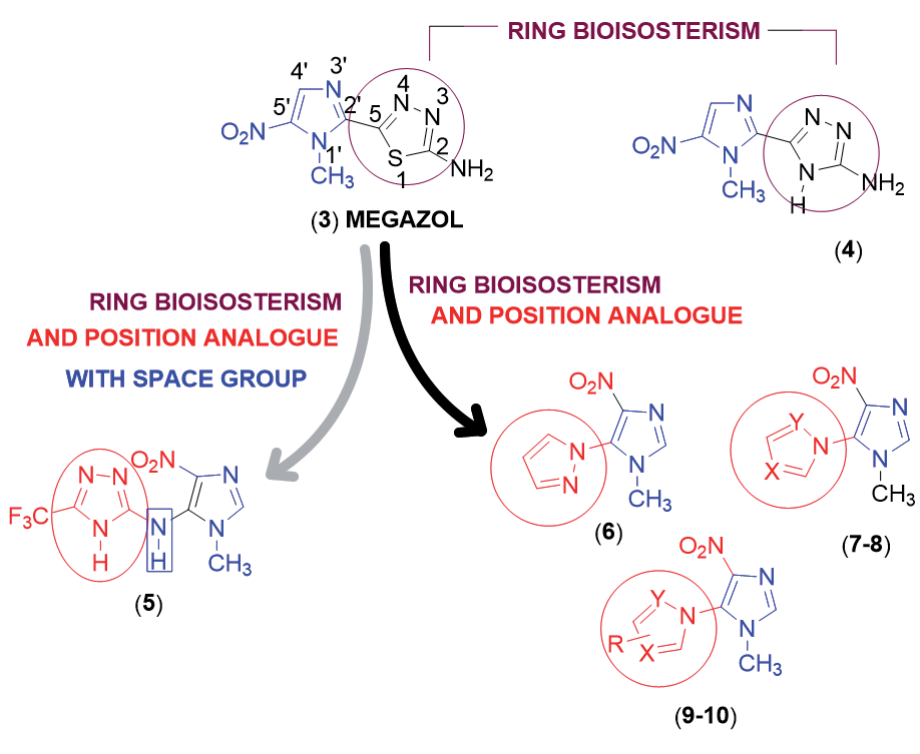
rational planning of nitroimidazole compounds (4-10) analogs to megazol (3).

The aim of the present study was to evaluate whether the genotoxic effect was abolished in the seven synthesised nitroimidazoles (4-10) analogs to 3, as well as to correlate this effect to their *in vitro* activity in *T. cruzi*. The *in vitro* alkaline comet assay (CA) and the *in vitro* cytokinesis-block micronucleus assay (CBMN) in whole human blood cells (WHBC) were employed as genotoxicity assays. The *in vitro* CA is a useful, fast screening system in mammalian cells that can be used in a test battery during drug development.^25^ It is widely used in basic research in the pharmaceutical and chemical industry^26^ to investigate genotoxic mechanisms as screening assay but it is not preconised for regulatory purposes.^27^ The single cell gel electrophoresis (SCGE) or the CA measures the migration of DNA from agarose-embedded cells in an electric ﬁeld; it detects initial DNA lesions as primary single-strand breaks (SSB) and double-strand breaks (DSB), DNA breaks resulting from spontaneous hydrolysis of adducts, and repair intermediates associated with abasic sites and DNA incisions.^28^ The CBMN is the standard genotoxicity test for the detection of clastogenic and aneugenic activities of chemicals and considered as a gold standard test in mutagenesis by the International Workshop on Genotoxicity Test Procedures.^29^ An OECD guideline (No. 487) for the *in vitro* mammalian cell micronucleus test has been published.^30^ Micronucleus (MN) can arise from acentric fragments via chromosome breakage (clastogenicity) or they may be whole chromosomes resulting from aneugenic events. Acentric fragments or whole chromosomes are not included in the main nucleus on cell division and manifest as small MN, almost one-third the size of the main nucleus. Therefore, the presence of MN indicates unrepaired chromosomal damage which is manifested after anaphase.^30^ Increased MN frequency in lymphocytes is a recognised predictor of cancer risk.^31^

## MATERIALS AND METHODS

The compounds 5-10 were prepared as previoulsly reported by our group.^19^ ^,^ ^22^ ^,^ ^32^ ^,^ ^33^

*In vitro trypanocidal assay* - To accomplishment of the experiments were used bloodstream trypomastigotes form Y strain of *T. cruzi*, obtained at the peak of the parasitemia (7th day after infection) of infected Swiss albino mice.^34^ The trypomastigotes underwent a differential centrifugation process for the separation of erythrocytes, leukocytes and concentration of the parasites in the plasma, and the purified parasites were resuspended in RPMI medium and cell concentration was determined by counting in the Neubauer chamber. Stock solution of each compound was prepared in dimethyl sulfoxide (DMSO), and the assays were performed in Dulbecco’s modified Eagle medium in 96-well plates. In the first well was placed twice the highest desired concentration of each compound, in a final volume of 200 μL, in the following wells were added 100 μL of medium supplemented with 10% foetal bovine serum (FBS) and 2% L-glutamine. Subsequently, 100 μL of parasite suspension (10^6^ trypomastigotes) were added, resulting in a final concentration of 5 x 10^6^ parasites/mL, incubated for 24 h at 37ºC under a 5% CO_2_ atmosphere and quantified in Neubauer’s chamber by light microscopy. The activity of the derivatives was expressed by the parameter which corresponds to the concentration of the compound that produces 50% lysis of the parasites (IC_50_). At least four independent experiments were performed and the mean and standard deviation were calculated. Benznidazole (1) was used as a reference drug. The experiments were performed in accordance with the guidelines established by the Oswaldo Cruz Foundation Committee of Ethics for the Use of Animals (L 038/2018).

*In vitro treatment and cytotoxicity assay in whole human blood cells (WHBC)* - Heparinised WHBC was obtained by venipuncture from healthy young non-smoking volunteers with no known recent exposures to genotoxic chemicals or radiation immediately before the assays. WHBC was treated for 2 h at 37ºC with different concentrations of compounds 3, 5, 8, 10 (149-10,000 µM) or 4, 6, 7, 9 (149-6,400 µM) in 5% (v/v) DMSO (solvent-control) and then used in the assays. The cytotoxicity assay aims to establish the degree of cell viability after treatment with nitroimidazoles to define the ranges concentrations to be tested in the *in vitro* CA. Cell viability was determined at the end of the treatment using the fluorescein diacetate (FDA)/ethidium bromide (EtBr)-assay, in which viable cells are labelled in green, while dead ones display orange-stained nuclei. WHBC (50 μL) was mixed with an equal volume of the freshly prepared staining solution consisting of 30 μg/mL FDA plus 8 μg/mL EtBr in phosphate-buffered saline (PBS). Samples (50 μL) were spread on a microscope slide and covered with a coverslip and observed using a fluorescence microscope. Two hundred cells were analysed for each treatment.^32^ The research project involving the use of human blood samples was approved by the Committee on Ethics in Research with Human Beings - CEP Fiocruz/IOC (CAAE: 41684815.3.0000.5248) under the consolidated opinion of CEP No. 1066061.

*In vitro alkaline comet assay (CA) in whole human blood cells (WHBC)* - DNA damage in WHBC was evaluated at the end of 2 h-treatment in duplicate with compounds 3 to 10, at the same concentrations indicated above using the *in vitro* CA. Methyl methane-sulfonate (MMS) (160 μM) (Sigma-Aldrich) was used as a positive control. Aliquots of 5 μL WHBC were mixed with 120 μL of 0.5% low melting-point agarose (LMPA) (Sigma-Aldrich) in PBS at 37ºC and were applied to microscope slides (with frosted ends), previously covered with 1.5% normal melting-point agarose (Sigma-Aldrich). Slides were prepared, lysed (pH 10; 4-5ºC) and processed as described earlier,^32^ using a time of alkali denaturation of 20 min and electrophoresis (0.86 V/cm and 300 mA) of 20 min at a pH > 13. After the neutralisation, fixation and staining steps^32^ the slides were analysed using a fluorescence microscope at 400 X magnification. Fifty randomly selected cells per slide (200 cells per treatment) stained with EtBr (20 µg/mL) were analysed visually according to tail size into one of four classes of DNA damage: 0 (undamaged, i.e., no visible tail), 1 (slightly damaged), 2 (moderately damaged) and 3 (maximally damaged, i.e, head of comet was very small and most of the DNA in the tail).^24^ ^,^ ^32^ The DNA damage was expressed as percentage of cells into four classes and as arbitrary units (AU) according to the formula: AU = (0 x *n*_0_) + (1 x *n*_1_) + (2 x *n*_2_) + (3 x *n*_3_), where *n =* the number of cells analysed in each class. The total DNA damage score in AU (TAU) for 200 cells can range from 0 TAU (200 undamaged cells) to 600 TAU (all cells maximally damaged). Differences between the mean values of TAU from two and three independent experiments under the same conditions, respectively, for each concentration of compounds 4-8,10 and 3, 9 were tested for significance (p < 0.05) in relation to the solvent-control group using Student’s one-tailed *t*-test. In addition, the effects of compounds 3-10 on the intercellular distribution of DNA damage were tested for statistical significance using one-way ANOVA followed by a Dunnett’s multiple comparison test to compare each concentration of the compounds.^32^ The computer program GraphPad Prism® sixth version was employed in the statistical analysis of the data.

*In vitro cytokinesis-block micronucleus assay (CBMN) with whole human blood cells (WHBC)* - Heparinised WHBC samples were obtained by venipuncture from volunteers as described above. The CBMN was performed with WHBC cultures following the OECD guideline 487.^30^ WHBC cultures were set up in 10 mL plastic culture tubes (Nunc, Denmark) by adding 0.5 mL freshly collected blood to 4.5 mL of pre-warmed (37ºC) 1640 RPMI medium (Gibco, USA) supplemented with 20% FBS (Gibco, USA), 10^2^ UI/mL penicillin G potassium, 10^2^ µg/mL streptomycin sulfate, 3% phytohaemagglutinin M (PHA-M: Gibco, USA) and incubated at 37ºC. WHBC cultures were treated for 4 h at 37ºC with different concentrations of compounds 3 to 10 (150-10,000 µM) in 5% (v/v) DMSO (solvent-control) 44 h after the start of the cultures. WHBC cultures exposed to mitomycin C (MMC) (1.0 µg/mL in water) (Bristol, USA) for 2 h or to N-deacetyl-N-methylcolchicine in water (Demecolcine: Sigma-Aldrich) 0.02 µg/mL for 28 h were used as positive control cultures. After the treatment, the WHBC cultures were centrifuged (900 rpm, 10 min) and washed with 5 mL PBS. After another centrifugation, the cell pellets were resuspended in 5 mL fresh complete RPMI medium as described above, but without PHA-M and with 4.5 µg/mL cytochalasin B (CytB) (Sigma-Aldrich) and incubated at 37ºC. CytB has cytokinesis-block activity leading to the formation of binucleate cells. Then, the cultures were harvested at the end of a total culture time of 72 h by centrifugation and treated with 5 mL hypotonic solution (0.56% KCl, 4-6ºC) for 10 min and fixed once for 10 min at room temperature with 5 mL methanol/glacial acetic acid (5:1, -20ºC) mixed with an equal amount of 0.9% NaCl and then fixed three times with methanol/glacial acetic acid (5:1, -20ºC) for 15 min at room temperature. The fixed cell suspension was dropped on a clean glass slide and the slide was air-dried on a heating plate (60ºC). Air-dried slides were stained with 60 µg/mL acridine orange in Sörensen buffer (0.03 M KH_2_PO_4_, 0.03 M Na_2_HPO_4_) for 3s, then they were embedded in distilled water and covered with a coverslip. MN showing bright green fluorescence were analysed using a fluorescence microscope at 400 X magnification. MN were scored in 2,000 binucleated cells (BNC) for culture and the number of the micronucleate cells (MNC) in 2,000 BNC was determined. Cytotoxicity was measured using the cytokinesis-block proliferation index (CBPI) and the replication index (RI) which were calculated from 500 cells. The CBPI indicates the average number of nuclei per cell, and may be used to calculate cell proliferation. The RI indicates the relative number of cell cycles per cell during the period of exposure to cytoB in treated cultures compared to control cultures and can be used to calculate the % cytostasis. CBPI was calculated according to the formula: CBPI = [(No. mononucleate cells) + (2 x No. binucleate cells) + (3 x No. multinucleate cells)] / *N*, where *N* indicates the total number of cells scored. RI (%) was calculated according to the formula:

$$RI\;\left(\%\right)=\frac{((No.\;binucleate\;cells)\;+\;(2\;x\;No.\;multinucleate\;cells))\;÷\;(Total\;number\;of\;cells)T}{((No.\;binucleate\;cells)\;+\;(2\;x\;No.\;multinucleate\;cells))\;÷\;(Total\;number\;of\;cells)C}\;x\;100$$

where T indicates treated cultures and C control cultures 

% cytostasis = 100 - RI

Cytotoxicity was evidenced in the occurrence of reduction in CBPI or RI of cultures treated by test substances when compared to control cultures. The measurement of cytotoxicity was used to select the concentrations of the test substance to be analysed for the presence of MN. The maximum concentration used in MN analysis recommended by OECD 487 in the presence of CytB is the one that induces 45 ± 5% reduction of CBPI or RI when compared to the solvent - control.^30^ However, it was adopted in this work as the maximum concentration in CBMN one that induced a proliferation inhibition not exceeding about 50% established by ICH S2 (R1) for lymphocyte cultures*.* ^35^

The chi-square test was performed from a contingency table tabulating the number of BNC with and without MN to test the significance (p < 0,05) of the results of each concentration of compounds 3-10 in relation to the solvent-control culture or of each concentration of positive controls in relation to untreated cultures (control culture). The chi-square test for trend was performed to analyse if the increase of MNC was concentration-related.^30^

## RESULTS

**Fig. 3:**
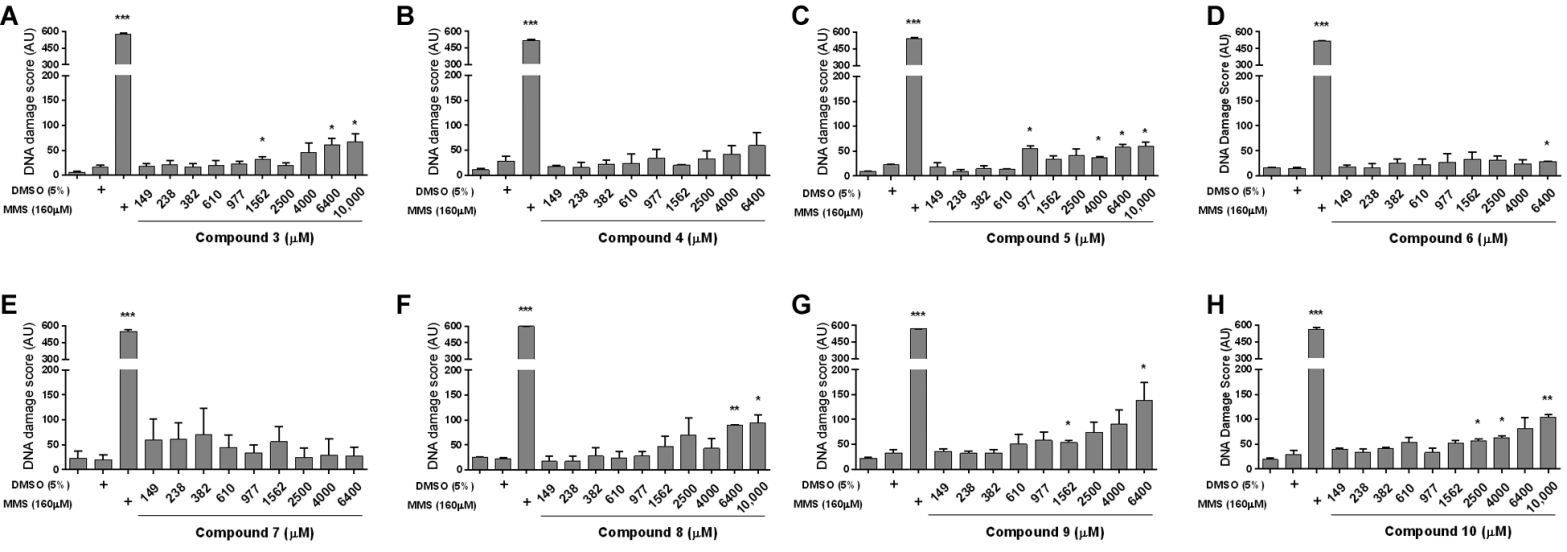
DNA damage induction by nitroimidazoles (3-10) in human whole blood cells using the *in vitro* alkaline comet assay. A: megazol (3) (n = 3); B: compound 4 (n = 2); C: compound 5 (n = 2); D: compound 6 (n = 2); E: compound 7 (n = 2); F: compound 8 (n = 2); G: compound 9 (n =3); H: compound 10 (n = 2). Results are expressed as mean ± standard error mean of total arbitrary units (AU) from two and three independent experiments, respectively for each concentration of compounds 4-8, 10 and 3,9. Only the result of compound 4 has already published (ref. 32). Control corresponds to untreated culture; solvent-control corresponds to 5% dimethyl sulfoxide and positive control to 160 µM methyl methane-sulfonate. For Student’s one-tailed *t-* test, the asterisks indicate significance at 5% (*) and 1% (**) levels in relation to the solvent-control and 0.1% (***) in relation to the control.

In addition to 3, the trypanocidal activity was tested against trypomastigote forms of *T. cruzi* (Table I). After 24 h of treatment, the compound 10 showed the highest activity with an IC_50_ = 5.4 ± 0.6 μM, about eight times higher than 9 (IC_50_ = 45.3 ± 4.0 μM), 48 times higher than 4 (IC_50_ 256.8 ± 53.0 μM), 65 times higher than 8 (IC_50_ = 353.7 ± 27.0 μM), at least 93 times higher than 5 (IC_50_ > 500 μM) and 6 (IC_50_ > 500 μM) and 370 times higher than **7** (IC_50_ > 2000 μM) (Table I). Compound 10 showed the highest activity, about two times higher than 3 (IC_50_ = 9.9 ± 0.8 µM) and compound 1 (IC_50_ = 8.8 ± 1.1 µM) that is used clinically. Compounds 4-9 were considered inactive molecules based on their IC_50_ values.^20^ Using the FDA/EtBr assay and *in vitro* CA, it was investigated the cytotoxicity of compounds 3-10 (Table II) and their capacity to induce DNA damage in WHBC (Fig. 3). Compound 8 (149-10,000 µM) was the only nitroimidazole that did not reduce cell viability. The treatment of WHBC with 3, 5 and 10 (range: 149-10,000 µM) and with 4, 6, 7 and 9 (149-6,400 µM) for 2h at 37ºC slightly reduced cell viability (lethality variation range: 1-4%) compared to solvent-control and this effect was not concentration dependent (Table II). All concentrations of the eight compounds studied showed acceptable levels of cytotoxicity, ie, they did not induce values greater than 30% decrease in cell viability when compared to the solvent-control and consequently used in the CA.^36^ The compounds 4^32^ and 7 did not cause DNA strand breaks in the range of 149-6,400 µM compared to the solvent-control group (p > 0.1). However, a significant (p < 0.05) genotoxic effect was observed at concentrations higher than to 610 µM for **5**, higher than to 977 µM for 3 and 9, higher than to 1,562 µM for 10, and highly significant (p < 0.01) at concentration higher than 4,000 µM (6,400 µM) and significant (p < 0.05) at 10,000 µM for 8. The genotoxic effect was concentration-dependent for these compounds (Fig. 3) and was not associated with cytotoxicity as shown in Table II. And for compound 6 it was showed a significant (p < 0.05) DNA damage only in the highest concentration tested (6,400 µM). The positive control, 160 μM MMS, induced an extremely significant (p < 0.001) genotoxic effect compared to the control group with a TAU mean value of 554.2 ± 9.9.

**TABLE I:** Effect of the nitroimidazoles (3-10) on trypomastigote forms of *Trypanosoma cruzi*

Compounds	IC_50_/24 h (µM)^*a*^
3	9.9 ± 0.8^*b*^
4	256.8 ± 53.0^*c*^
5	> 500
6	> 500
7	> 2000
8	353.7 ± 27.0
9	45.3 ± 4.0
10	5.4 ± 0.6
Benznidazole	8.8 ± 1.1

*a*: IC_50_: concentration that produces 50% lysis of the parasites; *b*: ref 23; *c*: ref 32.

**TABLE II:** Effect of nitroimidazoles on cytotoxicity assay in the human whole blood cells viability

	Compounds							
	3	4^*a*^	5	6	7	8	9	10
Control^*b*^	0.5	0	0	0	1.0	0	0	0
Solv-control^*c*^	6.0	0	0	2.0	0	0	0	0
149 µM	4.5	1.0	0	0	2.0	0	2.0	4.0
238 µM	10.0	1.0	0	4.0	0	0	2.0	1.0
382 µM	1.5	1.0	0	0	3.0	0	4.0	2.0
610 µM	5.5	0	0	0	2.0	0	0	0
977 µM	3.5	0	0	2.0	2.0	0	0	0
1,562 µM	3.0	1.0	0	0	0	0	0	0
2,500 µM	4.5	1.0	0	6	1.0	0	0	1.0
4,000 µM	6.0	0	2.0	0	0	0	0	0
6,400 µM	3.5	0	1.0	0	0	0	0	0
10,000 µM	2.5	-	2.0	-	-	0	-	2.0

*a*: ref 32; *b*: untreated culture; *c*: 5% dimethyl sulfoxide; results are expressed as percentage decrease (%) in cell viability.

In cells treated with 6,400 or 10,000 µM concentrations of compound 3, significant (p < 0.05) class 1 (22.5 % and 27.5%, respectively) of DNA damage was observed compared to the percentage of 4.2% showed by the solvent-control group. Compound 9 (6,400 µM) induced significant (p < 0.05) class 1 (40.7%) and highly significant (p < 0.01) class 2 (3.7%) damage compared to 9.0% and 0.7%, respectively for solvent-control. Compound 10 (10,000 µM) caused significant (p < 0.05) class 2 (2.5%) and class 3 (10.2%) damage compared to 0.2% and 2.2%, respectively for solvent-control. Compound 6 (6,400 µM) induced a significant increase of percentage of class 1 (10.5%) DNA damage in relation to 4.8% (solvent-control). In contrast, highly significant (p < 0.01) class 3 (87.7%) damage was observed after treatment with MMS (160 µM) compared to 0% for control culture.

Table III summarises the results of the CBMN with WHBC cultures exposed to different concentrations of compounds 3-10 (150-10,000 µM) for 4 h. With the exception of compound 5, the maximum concentrations used in MN analysis for compounds 3, 4, 6-10, that induced in the maximum 50% reduction of CBPI or RI in relation to solvent-control are indicated in the Table III. All concentrations of compound 5 were considered for MN analysis, because at the highest tested concentration (10,000 μM) there was only a 36% reduction of CBPI and 22% of RI below the maximum acceptable value of 50% reduction.^35^ The maximum concentration of 4,000 μM was established for compounds 3, 6 and 7; 1,600 μM determined for compounds 4 and 10 and 640 μM for compounds 8 and 9. Compounds 5, 6, 8-10 did not induce a significant increase in MN formation when compared to the solvent-control (p > 0.05 for 8 and 10; p > 0.1 for 5, 6 and 9) in the concentration range analysed. It was concluded that these five compounds did not induce chromosomal breaks and/or gain or loss of chromosomes in WHBC. In contrast, a highly significant mutagenic effect (p < 0.01) was observed at concentrations of 1,600 and 4,000 μM for compound 3, significant (p < 0.05) at 1,600 μM for compound 4 and at 4,000 μM for compound 7. When analysed by the chi-square test for trend, the increase in MN formation was concentration-dependent for compounds 3 (p < 0.001), 4 (p < 0.05) and 7 (p < 0.01). The positive control, MMC (1.0 μg/mL) caused an extremely significant clastogenic effect (p < 0.001) inducing in 2,000 BNC, 38 MNC compared to 13 MNC in the control culture and a significant effect (p < 0.05) with 56 and 40 MNC, respectively, compared at 32 and 20 MNC in the control culture. Demecolcine (0.02 μg/mL) showed a highly significant aneugenic effect (p < 0.01) inducing in 2,000 BNC, 37 and 42 MNC, respectively, compared at 13 and 20 MNC (control) and a significant effect (p < 0.05) with 30 MNC compared to 13 MNC in the control culture.

**TABLE III:** DNA damage induction by nitroimidazoles (3-10) in human whole blood cells using the *in vitro* cytokinesis-block micronucleus assay

Compounds	Groups	CBPI	RI (%)	NMNC/2000 binucleated cells	Chi-square-test (p)
3	Control^*a*^	1.322	-	13	-
	Solvent-control^*b*^	1.294	-	24	-
	150 µM	1.342	119	24	-
	640 µM	1.302	105	20	-
	1,600 µM	1.202	69	47	< 0.01 **
	4,000 µM	1.146	49	50	< 0.01 **
	10,000 µM	1.108	38	-	-
4	Control^*a*^	1.236	-	13	-
	Solvent-control^*b*^	1.200	-	25	-
	150 µM	1.220	106	28	-
	640 µM	1.230	107	36	-
	1,600 µM	1.218	76	44	< 0.05 *
	4,000 µM	1.054	22	-	-
	10,000 µM	1.052	23	-	-
5	Control^*a*^	1.188	-	32	-
	Solvent-control^*b*^	1.238	-	25	-
	150 µM	1.310	151	20	-
	640 µM	1.228	111	8	-
	1,600 µM	1.192	95	20	-
	4,000 µM	1.340	167	24	-
	10.000 µM	1.152	78	36	-
6	Control^*a*^	1.184	-	32	-
	Solvent-control^*b*^	1.128	-	35	-
	150 µM	1.184	148	32	-
	640 µM	1.204	132	12	-
	1,600 µM	1.134	114	16	-
	4,000 µM	1.068	56	12	-
	10,000 µM	1.036	24	-	-
7	Control^*a*^	1.172	-	32	-
	Solvent-control^*b*^	1.082	-	17	-
	150 µM	1.160	211	9	-
	640 µM	1.170	220	10	-
	1,600 µM	1.016	23	-	-
	4,000 µM	1.048	68	33	< 0.05 *
	10,000 µM	1.034	43	-	-
8	Control^*a*^	1.168	-	40	-
	Solvent-control^*b*^	1.200	-	36	-
	150 µM	1.154	150	20	-
	640 µM	1.108	69	16	-
	1,600 µM	1.028	26	-	-
	4,000 µM	1.020	17	-	-
	10,000 µM^*c*^	-	-	-	-
9	Control^*a*^	1.250	-	20	-
	Solvent-control^*b*^	1.160	-	36	-
	150 µM	1.188	105	24	-
	640 µM	1.102	60	20	-
	1,600 µM	1.034	17	-	-
	4,000 µM	1.022	11	-	-
	10,000 µM	1.060	31	-	-
10	Control^*a*^	1.208	-	32	-
	Solvent-control^*b*^	1.238	-	25	-
	150 µM	1.174	79	24	-
	640 µM	1.148	58	27	-
	1,600 µM	1.122	61	40	-
	4,000 µM	1.104	23	-	-
	10,000 µM	1.006	3	-	-

*a*: untreated culture; *b*: 5% dimethyl sulfoxide; CBPI: cytokinesis-block proliferation index; RI: replication index; NMNC: number of the micronucleate cells; *c*: high cytotoxicity did not allow the calculation of CBPI and RI; the maximum concentrations used in the analysis of micronucleus by inducing in the maximum 50% reduction of CBPI or RI relative to the solvent-control are indicated in bold; for the chi-square test, the asterisks indicate significance of the increase of the NMNC/ 2,000 binucleated cells at 5% (*) and 1% (**) levels in relation to solvent-control.

## DISCUSSION

In the present study it was evidenced for compound 3, a DNA strand break effect through the *in vitro* CA after 2 h treatment at the concentrations of 1,562, 6,400 and 10,000 μM in the WHBC. In the *in vitro* CA, the highest concentration (10,000 μM) employed corresponds to the maximum concentration to be tested *in vitro* for relatively non-cytotoxic substances.^37^ Similar results were obtained by Boechat et al.^24^ and Carvalho et al.^32^ who reported a highly significant genotoxic effect for the compound 3 (p < 0.01) on the same test system and experimental conditions for concentrations of 1,562 μM, 2,500 μM and 4,000 μM without reduction of cell viability in the tested range (380-4,000 μM). The *in vitro* CA performed on Vero cells, lymphocytes and whole blood^16^ was highly sensitive in detecting genotoxicity of 3 at concentrations in the range of 8.8 to 35 μM, well below those employed by Nesslany et al.^18^ with the same treatment period of 4 h. Poli et al.^16^ showed in fresh leucocytes from rats and mice a dose-response relationship of DNA damage induced by 3. Our results showed that 3 induced chromosomal breaks and/or gain or loss of chromosomes in human lymphocytes evidenced by the highly significant increase of MNC at concentrations of 1,600 and 4,000 μM in a concentration dependent manner after treatment period of 4h. The highest concentration of 3 (10,000 μM) was not evaluated for induction of MNs because it caused 63% of CBPI reduction and 62% of RI reduction, higher than the limit of 50% of cytotoxicity recommended for analysis by the ICH S2(R1).^35^ Nesslany et al.^18^ also reported for 3 provided by Farmanguinhos a high mutagenic activity in the *in vitro* micronucleus assay in L5178Y mouse lymphoma cells and treatment for 24 h at concentrations of 625 µM and 1,250 µM not associated to cytotoxicity. The compound 3 had its genotoxicity confirmed in other *in vitro* and *in vivo* mammalian cell assays. It was a potent inducer of structural chromosome aberrations *in vitro* in human lymphocytes after 4 h of treatment at the highest concentration possible to be analysed due to cytotoxicity (625 µM); significant increase at the three concentrations (156, 312 and 625 µM) for the treatment of 20 h and at 357 and 625 µM for the 44 h treatment. In the *in vivo* micronucleus assay in rat bone marrow cells, the two daily doses (two days) given orally (500 and 1,000 mg/kg), with 24 h sampling after the second dose induced a significant increase in frequency of micronucleated polychromatic erythrocytes in male and female Sprague-Dawley rats. Although 3 is a potent trypanomicidal and bioavailable agent when administered orally, its toxicity has led to the discontinuation of its development process for the treatment of Chagas’ disease and sleeping disease.^18^ This compound showed high trypanocidal activity *in vitro*, ie an IC_50_ = 9.9 ± 0.8 μM for *T. cruzi* and 0.14 ± 0.01 μM for *T. brucei*.^32^

With the exception of compound 3, which showed in the concentrations 1,562, 6,400 and 10,000 μM, DNA strand break inducer effect and in 1,600 and 4,000 μM clastogenic and/or aneugenic effect in WHBC, all compounds analogs to 3 that were positive in the *in vitro* CA (5,6,8-10) did not induce chromosomal breaks and/or gain or loss of chromosomes.The DNA strand breaks induced by the five analogs (5, 6, 8-10) of 3 in the CA may be repaired, resulting in no persistent effect, and may be lethal to the cell.^38^ On the other hand, compounds 4^33^ and 7 that did not induce DNA strand breaks were clastogenic and/or aneugenic in human blood cells, respectively at concentrations of 1,600 and 4,000 μM. Therefore, the clastogenic DNA damage of 4 and 7 was not detected by the *in vitro* CA.

In relation to compound 6, mutagenicity was reported in the Ames test in TA100 strain of *Salmonella typhimurium* at the highest tested concentration (50 μg/mL) in the absence and presence of S9 mixture.^39^ It showed cytotoxic activity in the concentration of 50 μg/mL in TA98 and in the range of 1.0-50 μg/mL in TA100 and TA1535 strains.^39^ Our results of the CBMN of 6 in WHBC, recommended by OECD (2016)^30^ differed from those obtained by Mello et al.^39^ who showed a significant increase of MNC in RAW 264.7 cells at concentrations of 10 and 100 μg/mL when treated for a period six times higher than that used in our study.

In the preclinical evaluation of drug candidates, genotoxicity tests are required by regulatory agencies to evaluate the potential risk of cancer induction. Among these tests the CA especially *in vivo*, the CBMN and the *in vivo* micronucleus assay are the most used in the evaluation of the potential risk of cancer induction.^40^ According to ANVISA, in agreement with the other internationally recognised regulatory agencies, it is recommended that genotoxicity tests should be completed prior to conducting phase 2 clinical trials.^41^

The CA allows the investigation of DNA damage in any cell culture or tissue that can be subjected to single cell isolation. Through this technique it is possible to evaluate DNA damage and repair in proliferating and non-proliferating cells at the individual level using extremely small cell samples (5-10 µL). The CA under highly alkaline conditions (pH > 13) during electrophoresis allowed the detection of a broader range of DNA damage.^28^ This includes SSB which may result from direct interaction of the test chemical with DNA or which are related to incomplete excision repair as well as alkali labile sites. As a result, not only clastogenic DNA damage can be detected but also lesions which may give rise to gene mutation.^38^

The presence of MN in lymphocytes indicates unrepaired damage, from consequences of chromosome mis-segregation or clastogenic events which is manifested after anaphase.^30^ Increased MN frequency in lymphocytes is a recognised predictor of cancer risk in humans and indicates pre-cancerous lesions.^31^

In the drug evaluation strategy performed, greater relevance should be given to the results obtained in the CBMN than in the *in vitro* CA because the former is considered the standard genotoxicity test in the guidelines for drug evaluation.^35^ ^,^ ^41^

It must also be considered that mutagenicity, clastogenicity and aneugenicity are the types of genotoxicity endpoints associated with human disease that should be given the most weight when conducting a human risk assessment. Assays evaluating DNA damage, such as DNA strand breaks in the CA and the measurement of DNA adducts can be useful to determine the presence of DNA damage and can be used to demonstrate an absence of strand breakage and therefore reduced potential to induce heritable alterations. However, their utility for quantitative evaluations is limited because the extent to which DNA damage may be repaired before conversion to a permanent genetic alteration is difﬁcult to ascertain. DNA strand breaks occur during DNA repair and during apoptosis and before necrosis, and so strand breakage may not always be related directly to the formation of mutations or chromosomal aberrations.^42^

Among the negative substances in the CBMN (5, 6, 8-10), substance 10 was the most promising to proceed with the development as a drug candidate in the treatment of Chagas disease. In addition to the absence of a cytogenetic damage effect inducing chromosomal breaks and / or gain or loss of chromosomes in human blood cells, substance 10 showed high trypanomicidal activity for *T. cruzi* (IC_50_ = 5.4 ± 0.6 μM), about two times higher than 3 (IC_50_ = 9.9 ± 0.8 μM)^23^ and 1 (IC_50_ = 8.8 ± 1.1 μM) used clinically. Substitution bioisosteric of 1,3,4-thiadiazole ring of 3 by lipophilic group linked to azole C-4 and the change from the 5-position nitro group to the 4-position of the imidazole ring in 10 abolished the undesirable mutagenic effect of prototype 3^9^ and as a consequence decreasing its effects on the carcinogenicity.^18^

As a follow-up test to evaluate metabolism, pharmacokinetics, and DNA repair of compound 10, the *in vivo* micronucleus assay for the detection of chromosome damage is recommended and performed in immature (polychromatic) bone marrow erythrocytes of mice or rats.^35^ ^,^ ^41^ And as a second *in vivo* genotoxicity assay to evaluate DNA strand breaks is recommended the *in vivo* CA especially in liver or stomach cells of rodents after oral exposure to compound 10.^35^ ^,^ ^38^ The use of both assays allows also evaluating the systemic or *in situ* genotoxicity.^40^

## References

[1] World Health Organization Chagas disease (American trypanosomiasis). The disease: what is
Chagas disease? 2018. http://www.who.int/chagas/disease/en/.

[2] Pérez-Molina JA, Molina I (2018). Chagas disease. Lancet.

[3] Dias JC, Ramos AN, Gontijo ED, Luquetti A, Shikanai-Yasuda MA, Coura JR (2016). 2nd Brazilian consensus on Chagas disease, 2015. Rev Soc Bras Med Trop.

[4] Pérez-Molina JA, Pérez-Ayala A, Moreno S, Fernández-González MC, Zamora J, López-Velez R (2009). Use of benznidazole to treat chronic Chagas' disease a systematic
review with a meta-analysis. J Antimicrob Chemother.

[5] Jackson Y, Alirol E, Getaz L, Wolff H, Combescure C, Chappuis F (2010). Tolerance and safety of nifurtimox in patients with chronic
Chagas disease. Clin Infect Dis.

[6] Pinazo MJ, Mun~oz J.Posada E.López-Chejade P.Gállego M.Ayala E (2010). Tolerance of benznidazole in treatment of Chagas' disease in
adults. Antimicrob Agents Chemother.

[7] Laboratório Farmacêutico do Estado de Pernambuco (2018). http://www.lafepe.pe.gov.br/category/benznidazol.

[8] Filardi LS, Brener Z (1982). Nitroimidazole-thiadiazole derivative with curative action in
experimental Trypanosoma cruzi infections. Ann Trop Med Parasitol.

[9] Bouteille B, Marie-Daragon A, Chauvière G, Albuquerque C, Enanga B, Dardé ML (1995). Effect of megazol on Trypanosoma brucei brucei acute and subacute
infections in Swiss mice. Acta Tropica.

[10] Enanga B, Keila M, Chauvière G, Dumas M, Bouteille B (1998). Megazol combined with suramin a chemotherapy regimen which
reversed the CNS pathology in a model of human African trypanosomiasis in
mice. Trop Med Int Health.

[11] Barrett MP, Fairlamb AH, Rousseau B, Chauviere G, Perie J (2000). Uptake of nitroimidazole drug megazol by African
trypanosomes. Biochem Pharmacol.

[12] Buschini A, Giordani F, de Albuquerque CN, Pellacani C, Pelosi G, Rossi C (2007). Trypanocidal nitroimidazole derivatives relationships among
chemical structure and genotoxic activity. Biochem Pharmacol.

[13] Salomão K, de Souza EM, Carvalho SA, Silva EF, Fraga CAM, Barbosa HS (2010). In vitro and in vivo activity of
1,3,4-thiadiazole-2-arylhydrazone derivatives of megazol on Trypanosoma
cruzi. Antimicrob Agents Chemother.

[14] Chauvière G, Bouteille B, Enanga B, de Albuquerque C, Croft SL, Dumas M (2003). Synthesis and biological activity of nitro heterocycles analogous
to megazol, a trypanocidal lead. J Med Chem.

[15] Ferreira RC, Ferreira LC (1986). Mutagenicity of CL 64855, a potent anti-Trypanosoma cruzi
drug. Mutat Res.

[16] Poli P, Mello MA, Buschini A, Mortara RA, de Albuquerque CN, da Silva S (2002). Cytotoxic and genotoxic effects of megazol, an anti-Chagas'
disease drug, assessed by different short-term tests. Biochem Pharmacol.

[17] Mital A (2009). Synthetic nitroimidazoles: biological activities and mutagenicity
relationships. Sci Pharm.

[18] Nesslany F, Brugier S, Mouries MA, Le Curieux F, Marzin D (2004). In vitro and in vivo chromosomal aberrations induced by
megazol. Mutat Res.

[19] Boechat N, Carvalho AS, Fernández-Ferreira E, Soares RO, Souza AS, Gibaldi D (2001). Novel nitroimidazoles with trypanocidal and cell growth
inhibition activities. Cytobios.

[20] Rosselli FP, Albuquerque CN, da Silva ABF (2005). Quantum chemical and statistical study of megazol-derived
compounds with trypanocidal activity. Int J Quantum Chem.

[21] Carvalho AS, Gibaldi D, Pinto AC, Bozza M, Boechat N (2006). Synthesis and trypanocidal evaluation of news
5-[N-(3-(5-substituted)-1,3,4-thiadiazolyl)]amino-1-methyl-4-nitroimidazoles. Lett Drug Des Discov.

[22] Carvalho AS, Menna-Barreto RFS, Romeiro NC, de Castro SL, Boechat N (2007). Design, synthesis and activity against Trypanosoma cruzi of
azaheterocyclic analogs of megazol. Med Chem.

[23] Carvalho SA, Silva EF, Santa-Rita RM, de Castro SL, Fraga CAM (2004). Synthesis and antitrypanosomal profile of new functionalized
1,3,4-thiadiazole-2-arylhydrazone derivatives, designed as non-mutagenic
megazol analogues. Bioorg Med Chem Lett.

[24] Boechat N, Carvalho AS, Salomão K, de Castro SL, Araujo-Lima CF, Mello FVC (2015). Studies of genotoxicity and mutagenicity of nitroimidazoles
demystifying this critical relationship with the nitro group. Mem Inst Oswaldo Cruz.

[25] Hartmann A, Elhajouji A, Kiskinis E, Poetter F, Martus H-J, Fjällman A (2001). Use of the alkaline comet assay for industrial genotoxicity
screening comparative investigation with the micronucleus
test. Food Chem Toxicol.

[26] Frötschl R (2015). Experiences with the in vivo and the in vitro comet assay in
regulatory testing. Mutagenesis.

[27] Collins AR (2015). The comet assay a heavenly method!. Mutagenesis.

[28] Singh NP, McCoy MT, Tice RR, Schneider EL (1988). A simple technique for quantitation of low levels of DNA damage
in individual cells. Exp Cell Res.

[29] Kirsch-Volders M, Sofuni T, Aardema M, Albertini S, Eastmond D, Fenech M (2003). Report from the in vitro micronucleus assay working
group. Mutat Res.

[30] Organisation for Economic Cooperation and Development OECD Guideline for the testing of chemicals. 487: in vitro
mammalian cell micronucleus test.

[31] Bonassi M, Znaor A, Ceppi M, Lando C, Chang WP, Holland N (2007). An increased micronucleus frequency in peripheral blood
lymphocytes predicts the risk of cancer in humans. Carcinogenesis.

[32] Carvalho AS, Salomão K, de Castro SL, Conde TR, Zamith HPS, Caffarena ER (2014). Megazol and its bioisostere 4H-1,2,4-triazole comparing the
trypanocidal, cytotoxic and genotoxic activities and their in vitro and in
silico interactions with the Trypanosoma brucei nitroreductase
enzyme. Mem Inst Oswaldo Cruz.

[33] Quaresma BMCS (2015). Síntese e avaliação tripanomicida e mutagênica de novos nitroimidazóis
substituídos com diferentes anéis azólicos.

[34] Silva LHP, Nussenzweig V (1953). Sobre uma cepa de Trypanosoma cruzi altamente virulenta para o
camundongo branco. Folia Clín Biol.

[35] International Conference on Harmonisation of Technical Requirements
for Registration of Pharmaceuticals for Human Use Guideline, guidance on genotoxicity testing and data
interpretation for pharmaceuticals intended for human use S2(R1). Approval
by the Steering Committee of S2(R1) under Step 4 and recommendation for
adoption to the three ICH regulatory bodies (9 November
2011). http://www.fda.gov/downloads/Drugs/Guidances/ucm074931.pdf.

[36] Henderson L, Wolfreys A, Fedy J, Bouner C, Windebank S (1998). The ability of the comet assay to discriminate between genotoxins
and cytotoxins. Mutagenesis.

[37] Tice RR, Agurell E, Anderson D, Burlinson B, Hartmann A, Kobayashi H (2000). Single cell gel comet assay: guidelines for in vitro and in vivo
genetic toxicology testing. Environ Mol Mutagen.

[38] Organisation for Economic Cooperation and Development OECD guideline for the testing of chemicals 489: in vivo
mammalian alkaline comet assay.

[39] Mello FVC, Carvalho AS, Bastos MM, Boechat N, Aiub CAF, Felzenszwalb I (2013). Evaluation of genotoxic effects of new molecules with possible
trypanocidal activity for Chagas disease treatment. ScientificWorld Journal.

[40] Araldi RP, Melo TC, Mendes TB, de Sá Junior PL.Nozima BH.Ito ET (2015). Using the comet and micronucleus assays for genotoxicity studies
a review. Biomed Pharmacother.

[41] Agência Nacional de Vigilância Sanitária (2013). Guia para condução de estudos não clínicos de toxicologia e
segurança farmacológica necessários ao desenvolvimento de
medicamentos. GESEF.

[42] Mac Gregor JT, Frötschl R, White PA, Crump KS, Eastmond DA, Fukushima S (2015). IWGT report on quantitative approaches to genotoxicity risk
assessment II Use of point-of-departure (PoD) metrics in de?ning acceptable
exposure limits and assessing human risk. Mutat Res Genet Toxicol Environ Mutagen.

